# Evolutionary inactivation of a sialidase in group B *Streptococcus*

**DOI:** 10.1038/srep28852

**Published:** 2016-06-29

**Authors:** Masaya Yamaguchi, Yujiro Hirose, Masanobu Nakata, Satoshi Uchiyama, Yuka Yamaguchi, Kana Goto, Tomoko Sumitomo, Amanda L. Lewis, Shigetada Kawabata, Victor Nizet

**Affiliations:** 1 Department of Oral and Molecular Microbiology, Osaka University Graduate School of Dentistry, Suita, Osaka, Japan; 2 Department of Pediatrics School of Medicine, University of California San Diego, La Jolla, CA, USA; 3 Department of Urology, Nagoya University Graduate School of Medicine, Nagoya, Aichi, Japan; 4 Department of Molecular Microbiology and Ob/Gyn, Washington University School of Medicine, St. Louis, Missouri, USA; 5 Skaggs School of Pharmacy and Pharmaceutical Sciences, University of California San Diego, La Jolla, CA, USA; 6 Rady Children’s Hospital, San Diego, CA, USA

## Abstract

Group B *Streptococcus* (GBS) is a leading cause of bacterial sepsis and meningitis in newborns. GBS possesses a protein with homology to the pneumococcal virulence factor, NanA, which has neuraminidase (sialidase) activity and promotes blood-brain barrier penetration. However, phylogenetic sequence and enzymatic analyses indicate the GBS NanA ortholog has lost sialidase function – and for this distinction we designate the gene and encoded protein *nonA*/NonA. Here we analyze NonA function in GBS pathogenesis, and through heterologous expression of active pneumococcal NanA in GBS, potential costs of maintaining sialidase function. GBS wild-type and Δ*nonA* strains lack sialidase activity, but forced expression of pneumococcal NanA in GBS induced degradation of the terminal sialic acid on its exopolysaccharide capsule. Deletion of *nonA* did not change GBS-whole blood survival or brain microvascular cell invasion. However, forced expression of pneumococcal NanA in GBS removed terminal sialic acid residues from the bacterial capsule, restricting bacterial proliferation in human blood and *in vivo* upon mouse infection. GBS expressing pneumococcal NanA had increased invasion of human brain microvascular endothelial cells. Thus, we hypothesize that *nonA* lost enzyme activity allowing the preservation of an effective survival factor, the sialylated exopolysaccharide capsule.

*Streptococcus agalactiae* (Group B *Streptococcus*, GBS) is a Gram-positive bacterial pathogen that is a leading cause of sepsis, pneumonia, and meningitis during neonatal period and up to the first 90 days of life^1^,^2^. Each of the 10 different GBS capsular polysaccharide types^1^, though possessing different repeating subunits, share a terminal α-2-3-linked sialic acid (*N*-acetylneuraminic acid, Neu5Ac motif), which is identical to a sugar epitope capping many surface glycans on all mammalian cells^3^. Humans in particular express just the terminal α-2-3-linked Neu5Ac since they have lost the gene required to synthesize the alternative sialic acid, *N*-glycolylneuraminic acid (Neu5Gc) present in other mammals including primates^3^. The GBS sialylated capsule mimics a common presentation of Neu5Ac in the α-2-3-linkage, which contributes to evasion of the host immune system and promoting bacterial survival *in vivo*^4^. GBS capsular sialylation interferes with the host complement system to block C3b deposition and limit C5a deposition^5^,^6^, and inhibits neutrophil activation through interaction with inhibitory sialic acid-binding immunoglobulin-like lectin-9 (Siglec-9)^7^. The *in vivo* significance of these findings was corroborated in mice with and without Siglec-E, the closest homolog of human Siglec-9, which interacts with GBS in a sialic acid-dependent manner, triggering protein tyrosine phosphatase, SHP-1, recruitment to its intracellular domain and suppressing myeloid cell inflammatory responses^8^.

*Streptococcus pneumoniae* (pneumococcus) is a related Gram-positive pathogen and a major cause of pneumonia, sepsis, and meningitis^9^,^10^. Most severe *S. pneumoniae* diseases occur in children younger than 2 years and adults older than 65 years. The polysaccharide capsule of *S. pneumoniae* confers the antigenicity utilized to classify *S. pneumoniae* into at least 97 serotypes^11^. In contrast to GBS, no *S. pneumoniae* strains express sialic acid in its capsular polysaccharide. Instead, the bacterium expresses three sialic acid-cleaving enzymes or sialidases, NanA, NanB, and NanC^11^,^12^. The *nanA* and *nanB* genes are located in the same operon and detected in almost all clinical isolates, whereas the *nanC* gene is present in approximately half (51%) of isolates^12^. While the molecular functions of NanB and NanC in the pathogenesis are unclear, NanA has been identified as a multifunctional protein contributing to pneumococcal virulence^13^,^14^. NanA is a cell-wall-anchored protein and works as an invasin into human brain microvascular endothelial cells (hBMEC) through its LamG superfamily domain^14^,^15^,^16^. An isogenic *S. pneumoniae* Δ*nanA* mutant strain showed >90% reduction in adhesion and invasion efficiency compared to its parent strain; complementation of NanA expression on a plasmid vector restored the adherence/invasion phenotype. Furthermore, heterologous expression of NanA in *Lactococcus lactis* conferred an adhesion and invasion frequency ~10-fold greater than empty-vector-transformed control^14^. The NanA LamG domain induces inflammatory cytokine production from the brain endothelial cells, and the resulting cell activation promotes pneumococcal internalization^16^. In addition, desialylation of leukocyte cell surfaces by NanA resulted in MAP kinase phosphorylation and NF-κB activation through unmasking of Siglec-5^17^.

Here, we identify through homology searching a putative ortholog of pneumococcal NanA that is present in GBS strains. The biological consequences for GBS of possessing a potential sialidase enzyme, while simultaneously expressing a sialylated capsule as an essential virulence determinant, were initially unclear. Our bioinformatics analysis suggested that, unlike pneumococcal NanA, the GBS orthologue has lost the LamG domain and cell wall-anchoring motif, and that there was a nonsense mutation in this gene in some GBS strains. Codon-based selection analysis indicated that pneumococcal *nanA* was under stronger negative selection than *nonA*. We find that the GBS strains do not possess neuraminidase activity, and for this distinction we designate the gene and encoded protein *nonA*/NonA. In contrast to earlier published findings with pneumococcal NanA mutants^13^,^14^,^16^, targeted deletion of the *nonA* gene in GBS did not alter resistance to human whole blood killing, brain microvascular endothelial cell invasion, or animal virulence. However, forced expression of active pneumococcal NanA in the GBS Δ*nonA* mutant removed terminal sialic acid from the GBS polysaccharide capsule, reducing GBS survival in whole blood, while promoting GBS invasion of brain microvascular endothelial cells. Taken together, our results strongly suggest that the loss of function as a sialidase in GBS NonA in contemporary GBS strains allowed the organism to preserve the selective advantage of sialylated capsule.

## Results

### Evolutionary analysis of a GBS *nanA* ortholog

**Figure 1:**
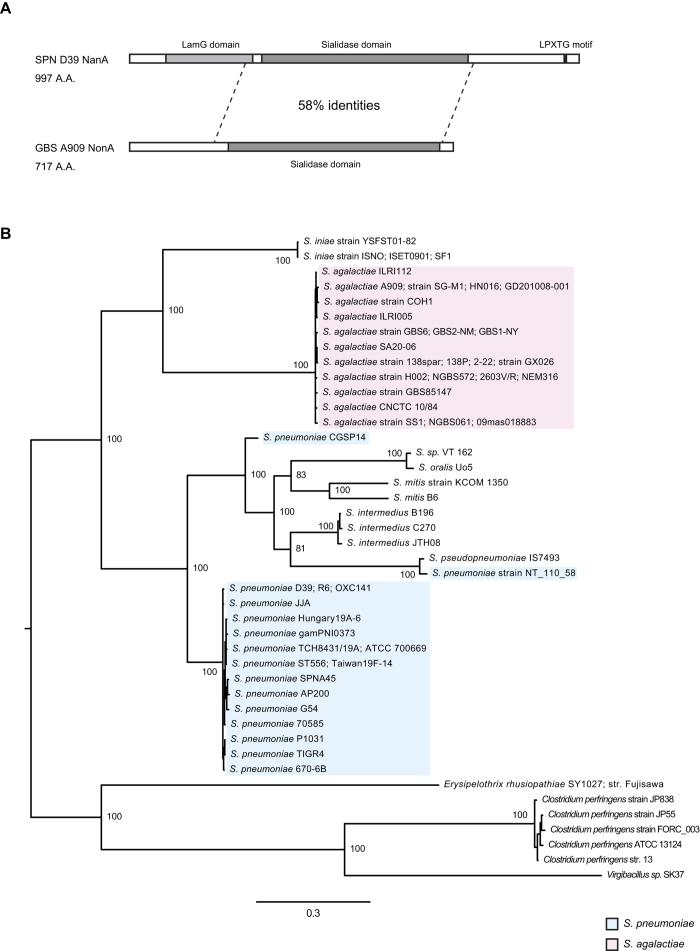
Phylogenetic analysis of *nanA* orthologs. (**A**) Schematic illustration of domains in *S. pneumoniae* NanA and GBS NonA. NonA lacks LamG domain and LPXTG motif conserved in NanA. (**B**) Bayesian phylogenetic tree of the *nanA* and *nonA* genes. The information on bacterial strains is listed in Supplementary Table 2. Strains with identical sequences are listed on the same branch. Percentage of posterior probabilities is shown near the nodes. The scale bar indicates nucleotide substitutions per site. *S. pneumoniae nanA* and GBS *nonA* genes are shaded in blue and red, respectively.

**Figure 2:**
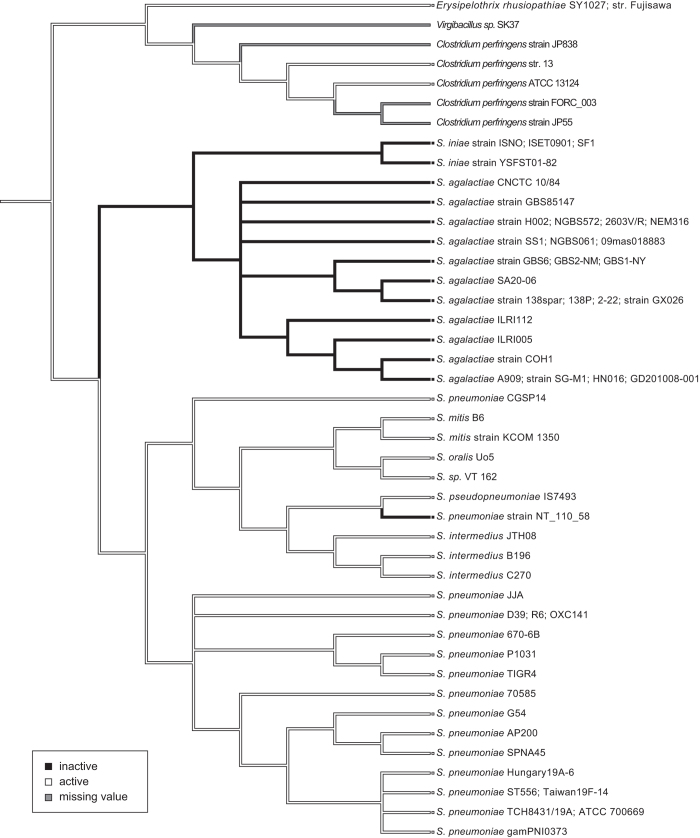
Ancestral state reconstructions based on the Bayesian phylogenetic tree. Parsimony reconstruction using Mesquite for active or inactive sialidase phenotypes is shown as white or black lines, respectively. Gray lines indicate missing values.

We performed a bioinformatics analysis on the *nonA* gene, an ortholog of pneumococcal *nanA* (*SAK_RS09520* or *SAK_1891*), annotated in the published genome of GBS strain A909. Amino acid sequence alignment analysis showed that SAK_RS09520 contains a sialidase domain but lacks the conserved lectin like-domain LamG and cell wall anchoring motifs present in pneumococcal NanA (Fig. 1A). GBS NonA shared 58% amino acid sequence identity with pneumococcal NanA across the sialidase domain, with lesser degrees of sequence identity with pneumococcal NanB and NanC (27–28% amino acid sequence identities) (Supplementary Table 1). Next, tBLASTn analysis revealed that a subset of species in the genus *Streptococcus* contains *nanA* orthologs and a phylogenetic analysis was performed using orthologous bacterial *nanA* sequences. Both Bayesian- and maximum likelihood phylogenetic analyses of these orthologs revealed similar patterns of genetic classification with high posterior probabilities or bootstrap values (Fig. 1B, Supplementary Fig. 1 and Supplementary Table 2). The sialidase genes of Gram-positive and rod-shaped bacteria, *Erysipelothrix rhusiopathiae, Clostridium perfringens, and Virgibacillus sp.,* were used to root, since with the exception of homologous genes from other streptococci, these genes exhibited the highest similarity with pneumococcal *nanA. E. rhusiopathiae* and *C. perfringens* are known to produce active sialidases^18^,^19^,^20^. Both trees indicate that the *nanA* ortholog genes of *S. mitis* and *S. pseudopneumoniae* diverged from each other, having shared a common ancestor. Of note, *nanA* genes of *S. pneumoniae* strains CGSP14 and NT_110_58 were distinct from those of other pneumococcal strains. The phylogenetic analysis revealed that the GBS *nonA* represents a single lineage in a cluster otherwise composed of *S. iniae,* which is a pathogen of fish and occasional nosocomial infections in humans^21^. NonA of both *S. iniae* and GBS lack an LPXTG motif, which is conserved in the NanA proteins of other streptococcus species. All streptococcal sialidases except GBS NonA possess the LamG domain (Fig. 1A and Supplementary Fig. 2). Further analysis of the genome database indicates that five GBS strains (GX026, SA20-06, 2-22, 138spar, and 138P) carry a *nonA* gene containing a nonsense mutation (Supplementary Table 3). In addition, we measured bacterial sialidase activities using streptococcal type strains and clinical isolates (Supplementary Fig. 3). Type strains of *S. oralis, S. intermedius,* and *S. pseudopneumoniae* showed positive sialidase activities. In contrast, the sialidase activity of *S. mitis*, GBS, and *S. iniae* strains was always below the detection limit. Previously, Killian *et al*. reported that 100% of 17 *S. pneumoniae* and 3 *S. pseudopneumonia*e strains and 69% of 54 *S. mitis* strains showed positive sialidase activity^22^. Some *S. mitis* strains appear to have reduced the genome sizes and may have lost virulence-associated factors including NanA in a reductive evolutionary process^23^,^24^. Thus, it is likely that *S. mitis* strains exhibit a diversity of sialidase activity. Furthermore, the result of an ancestral reconstruction technique suggests the possibility that sialidase activity was lost in the *nonA* lineage rather than gained in the *nanA* lineage (Fig. 2). Together these results suggest that streptococcal *nanA* orthologs diverged into two major groups, one consisting of *S. mitis, S. intermedius* and *S. pneumoniae*, and the other consisting of GBS and the *S. iniae* group. In the *S. iniae*/GBS group NonA appears to have lost its functional role.

**Figure 3:**
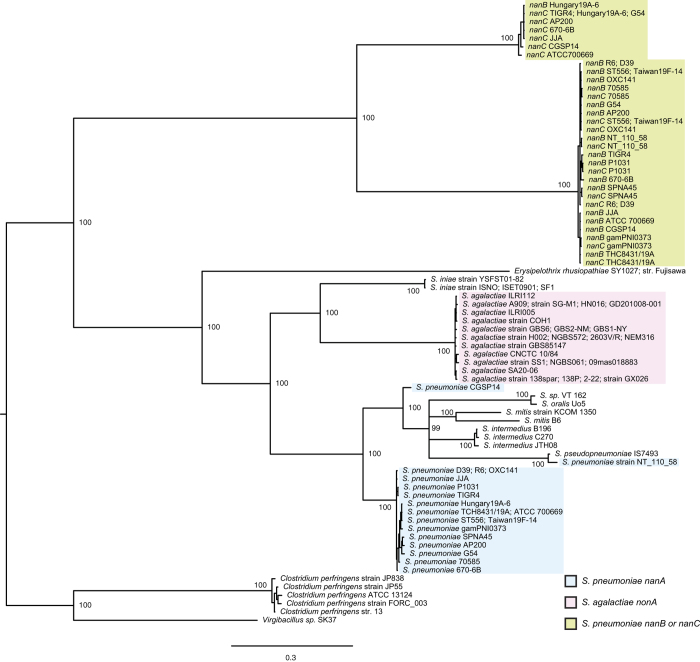
Bayesian phylogenetic tree of nanA, nanB, nanC, and GBS *nonA* genes. Percentage of posterior probabilities is shown near the nodes. Strains with identical sequences are listed on the same branch. The scale bar indicates nucleotide substitutions per site. Blue shows pneumococcal *nanA* and green is *nanB* or *nanC.* GBS *nonA* is shown as red.

To examine the relationship of pneumococcal *nanA, nanB, nanC,* and GBS *nonA*, a phylogenetic analysis was performed using the genes. Bayesian- and maximum likelihood phylogenetic analyses of the genes revealed similar patterns of genetic classification with high posterior probabilities or bootstrap values (Fig. 3 and Supplementary Fig. 4). The pneumococcal *nanB/nanC* were well separated from pneumococcal *nanA* or GBS *nonA*. We performed an additional evolutionary analysis on *nanA*, *nonA*, *nanB, nanC, bgaA, and strH* genes. BgaA and StrH, another pneumococcal exoglycosidases, remove galactose that is β1-4 linked to *N*-acetylglucosamine, and *N*-acetylglucosamine that is β1 linked to mannose, respectively^13^. Selection analysis through non-synonymous/synonymous ratio calculations by Fixed Effects Likelihood (FEL) and Fast, Unconstrained Bayesian AppRoximation (FUBAR) analyses suggested similar results. There were more codons evolving under negative selection in the *nanA* genes of *S. pneumoniae* strains (Table 1, Supplementary Table 4 and Supplementary Figs 5–10). In contrast, fewer codons evolving under negative selection were detected in the *nonA* genes of GBS as well as the *nanB* and *nanC* genes. Similar results were obtained with the *bgaA* and *strH* genes, indicating that *nanA* is under strong selective pressure. On the other hand, there were no or very few codons that appear to be evolving under positive selection in these genes. We conducted a likelihood ratio test to investigate whether pneumococcal *nanA* and GBS *nonA* genes have the same distribution of substitution rates across sites (Table 2). The distributions of substitution rates indicate no significant differences in between pneumococcal *nanA* and GBS *nonA* genes. However, there was a significant difference in selective regimes (dN/dS and proportions), especially in the proportions of codons under selection. These results suggest that a functional change of NanA would be deleterious in *S. pneumoniae*. In fact, pneumococcal NanA is a multifunctional protein, that promotes bloodstream survival^17^ and penetration of host endothelial cell barriers system^14^. In contrast to pneumococcal NanA, the GBS NonA does not appear to be under strong selective pressure, which supports our hypothesis that NonA no longer functions in GBS.

### Forced expression of NanA in GBS degrades terminal sialic acids of its capsule

**Figure 4:**
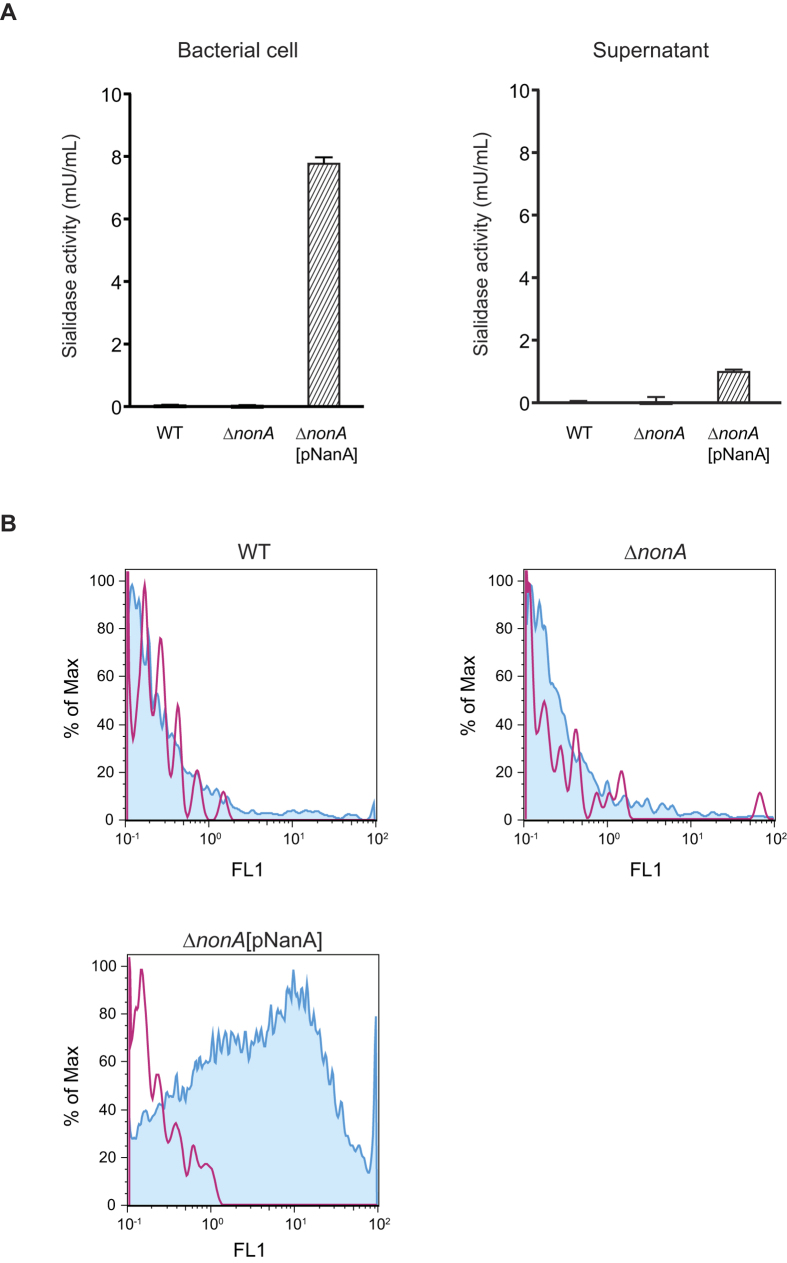
NanA degrades terminal sialic acid displayed on GBS polysaccharide capsule. (**A**) Sialidase activities of GBS cells and culture supernatant. After 2 h incubation at 37 °C, fluorescence of sialidase-degraded substrate was measured with excitation and emission wavelengths of 350 and 460 nm, respectively. Data are presented as the mean of sextuplets samples. S.E. values are represented by vertical lines. The sensitivity is 0.3 mU/mL. (**B**) FITC-labeled ECA binding to live GBS. Red line and blue histogram represents data for bacterial strains incubated without or with ECA, respectively.

To investigate the role of NonA in bacterial pathogenesis, we constructed an isogenic GBS Δ*nonA* mutant strain and then complemented the Δ*nonA* strain with the functional pneumococcal NanA as described in the Methods section. The expression of the *nonA* gene in a GBS wild-type (WT) strain was higher than that of the well-characterized *cylE* gene encoding the GBS β-hemolysin/cytolysin (Supplementary Fig. 11). Sialidase activities of GBS WT, Δ*nonA,* and Δ*nonA*[pNanA] strains were determined using a fluorometric sialidase assay (Fig. 4A). Neither the WT nor Δ*nonA* GBS strains showed sialidase activity associated the bacterial cells or culture supernatants, but sialidase activity could be detected with heterologous expression of the pneumococcal enzyme.

We next investigated whether heterologous expression of a functional sialidase (NanA) would degrade the terminal sialic acid moiety on the GBS capsular polysaccharide repeating unit by flow cytometry with FITC-labeled *Erythrina cristagalli* agglutinin (ECA; Fig. 4B). ECA binds to terminal (unsialylated) galactose and the ECA binding level inversely reflects the level of sialylation on the GBS capsule. GBS WT and Δ*nonA* mutant strains showed similar histogram patterns and did not interact with the FITC-labeled ECA. On the other hand, the complemented GBS Δ*nonA*[pNanA] strain showed substantially higher fluorescence intensity when incubated with FITC-labeled ECA as compared to the strains incubated without FITC-labeled ECA. These results indicated that the GBS WT strain possessed no sialidase activity and the forced expression of the active sialidase in GBS could have the effect of degrading its own terminal sialic acid, a known immune evasion virulence factor of the pathogen with anti-complement, anti-phagocytic, and immunosuppressive properties^4^,^5^,^6^,^7^,^8^.

### NonA does not contribute to GBS invasion into hBMECs

**Figure 5:**
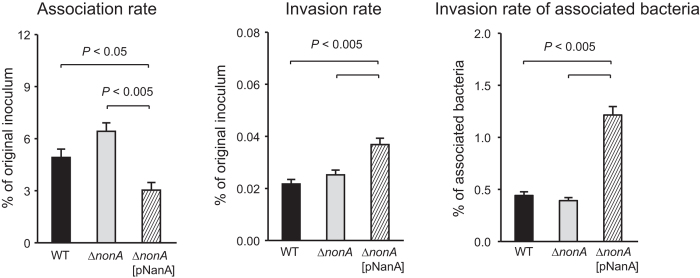
Rate of GBS adhesion to and invasion of hBMECs. GBS strain A909 and its isogenic mutant strains were examined for their adhesion and invasion activities. Adhesion rates were calculated by dividing CFU at 1 h after infection by CFU of original inoculum. Invasion rates were calculated by dividing CFU at 1 h after antibiotic addition by CFU of original inoculum. Data are presented as the mean of sextuplets samples. S.E. values are represented by vertical lines.

To examine the role of GBS NonA compared to the previously established role of pneumococcal NanA in the invasion of blood-brain barrier endothelium, we performed adherence/invasion assay using human brain microvascular endothelial cells (hBMECs) (Fig. 5). To quantify bacterial invasion, hBMECs were incubated with GBS strains for 1 hour (h) and further incubated for 1 h in medium containing antibiotics. WT GBS and the Δ*nonA* mutant did not differ in their adherence or invasion phenotypes to hBMEC (Fig. 5). The association of the Δ*nonA*[pNanA] strain was decreased as compared to that of other strains; however, invasion of the Δ*nonA*[pNanA] strains into human brain microvascular endothelial cells were significantly higher than that of GBS WT and Δ*nonA* strains. These results indicated that pneumococcal NanA, but not the endogenous GBS NanA homologue, can contributes to bacterial invasion of brain endothelial cells.

### Expression of sialidase in GBS inhibits its survival

**Figure 6:**
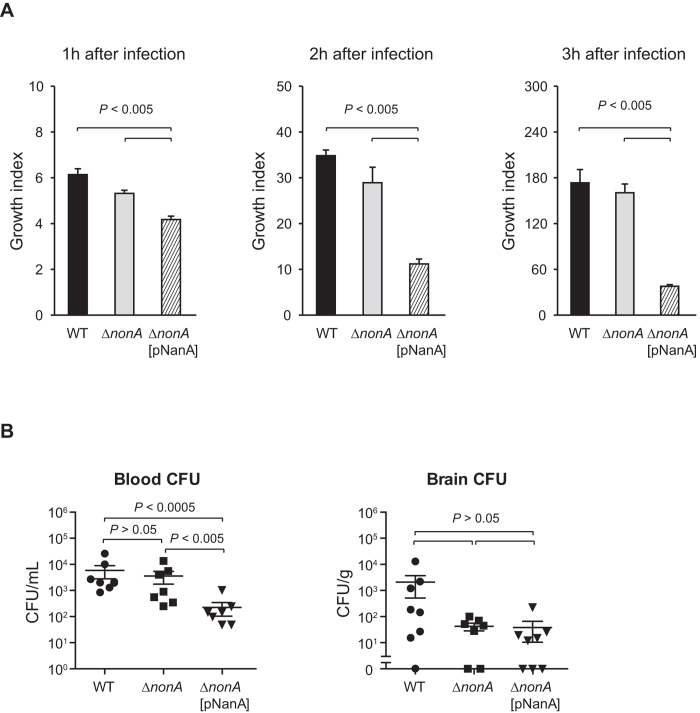
NanA expression diminishes GBS survival in human blood and mouse. (**A**) Growth of GBS strains in fresh human blood was determined. Bacterial cells were incubated in blood for 1, 2, and 3 h at 37 °C in a 5% CO_2_ atmosphere. Next, the mixture was serially diluted and plated on THY agar. Following incubation, the number of CFU was determined. Growth index was calculated by dividing CFU after incubation by CFU of original inoculum. Data are presented as the mean of sextuplets samples. S.E. values are represented by vertical lines. (**B**) Mice were infected with ~3.5 × 10^7^ CFU of GBS WT, *∆nonA,* or *∆nonA*[pNanA]. Blood and brain were collected at 20 h after infection. All mice were perfused with PBS prior to brain isolation. S.E. were represented by vertical lines. The difference between groups was analyzed using an Mann-Whitney *U*-test.

We compared bacterial survival rate *ex vivo* in human blood to compare the function of GBS NonA and active pneumococcal NanA in the GBS background (Fig. 6). The series of GBS strains were mixed with freshly collected human blood, and the mixture incubated for 3 h. Viable cell counts were determined hourly by plating diluted samples onto THY agar. No significant change in survival was noted comparing the isogenic Δ*nonA* mutant to the parent GBS WT strain. However, when NanA was introduced in this background, survival was approximately 72–79%, 39–44%, and 23% of WT levels after 1, 2, and 3 h, respectively. These results indicated that forced expression of NanA inhibits the survival of GBS in human blood, likely through the degradation of its terminal sialic acid on the capsule.

Finally, to investigate the potential role of NonA vs. an active sialidase in GBS pathogenesis, we infected GBS strains in mice intravenously, and compared bacterial CFU in blood and brains from mice 20 h after infection. Recovered CFUs of WT and Δ*nonA* strains in mouse blood were almost same. However, the Δ*nonA*[pNanA] expressing sialidase activity had on 14% the level of WT survival in the blood, consistent with the findings in the human blood survival assay.

## Discussion

*S. pneumoniae* contains three sialidases, NanA, NanB, and NanC. NanB works as a virulence factor in pneumococcal infection and NanC catalyzes intermediate metabolic compounds which acts as sialidase inhibitor^25^,^26^. The *nanC* gene was reported to significantly associate with clinical isolates from invasive diseases^12^,^27^. Our evolutionary analysis indicated that ~15% of the codons in the pneumococcal *nanA* gene evolved under negative selection, while few codons of *nanB* and *nanC* evolved in positive or negative selection. The results suggest that selection pressures exist such that the key enzymatic functions of NanA may not change. In contrast to the conservations on *nanA* in *S. pneumoniae*, the GBS NanA homologue (NonA) appears to have lost its sialidase activity. Sialyltransferases are highly conserved in GBS strains of each serotype when compared to other glycosyltransferase genes in a same operon, and the difference in genetic diversity support a hypothesis that sialic acid is critical for GBS survival in the human host^28^. Our results showed that restoration of an active sialidase function inhibited GBS survival in human blood *ex vivo* and mouse blood *in vivo*. Therefore, sialidase activity would be deleterious to the fitness of GBS, and GBS *nonA* appears to be a non-functional gene.

We recently reported a similar relationship between bacterial capsule and glycosidase in another pathogenic streptococci, group A *Streptococcus* (GAS, *Streptococcus pyogenes*)^29^. Almost all serotypes of GAS express a hyaluronan exopolysaccharide capsule and contain an inactivated version of the hyaluronidase (HylA) with a single nucleotide mutation resulting in Asp to Val substitution at amino acid position 199^30^. However, serotype M4 strains express an active HylA, while lacking hyaluronan capsule biosynthesis operon. The operon was predicted to represent a more recent evolutionary acquisition in most serotypes. Although hyaluronan capsule is a major GAS virulence factor, heterologous expression studies to generate partial encapsulation of M4 wild-type strain and full encapsulation of an isogenic mutant Δ*hylA* strain did not increase virulence. In this human bacterial pathogen, the conflicts between polysaccharide capsule and glycosidase would exert conflicting selective pressures, and resulted in mutual exclusivity. In the present work, we find a similar mutual exclusivity between sialidase activity and the GBS polysaccharide capsule.

It is widely thought that pathogenic microbes may explain some human polymorphisms^31^,^32^. Sialylated pathogens can dampen the immune response through interaction with Siglecs, and this molecular mimicry is considered to be one of the primary forces in the rapid evolution of human Siglecs^4^,^33^,^34^,^35^. For example, Siglec-13 and -17 may have been genetically eliminated during hominid evolution, because of interactions with pathogenic bacteria, including GBS, that cause invasive infections^33^. In addition, Siglec-14 and -5 expressed on neutrophils and monocytes appear to have evolved to provide a balanced response to pathogens and infants with Siglec-14 deficiency were the most prone to GBS immune subversion^36^. Thus, there exists a multifaceted interaction between pathogen and human evolution at the molecular level. The synergy of evolutionary bioinformatics and functional analysis may help to investigate the interplay between pathogen and host within an evolutionary framework and to identify new genetically stable therapeutic targets within pathogens and/or their human hosts.

## Methods

### Phylogenetic and evolutionary analysis

Phylogenetic and evolutionary analyses were performed as previously described with minor modifications^37^. Homologues of *nanA* were searched for using tBLASTn of NCBI BLAST^38^. Sequences from complete genomes with e-values <2 × 10^−85^ and >40% query coverage were selected for phylogenetic tree analysis. The sequences were aligned using MAFFT ver. 7.221 with FFT-NS-i strategy^39^ and edited using by Jalview^40^. Regions coding sialidase domain were used for further phylogenetic analysis. Edited sequences were aligned again using MAFFT with L-INS-i strategy. The best-fitting codon evolutionary models for maximum likelihood and Bayesian phylogenetic trees were determined by Kakusan4^41^. Maximum likelihood phylogenetic trees with bootstrap values were generated by RAxML ver. 8.1.20^42^. To validate phylogenetic inferences, Bayesian Markov chain Monte Carlo (MCMC) analyses were performed in MrBayes ver. 3.2.5^43^, sampling 10^6^ generations with a confirmation that the standard deviation of split frequencies was <0.01. Phylogenetic trees were drawn by FigTree ver. 1.4.2 (http://tree.bio.ed.ac.uk/software/figtree/) based on calculated data.

Tests for evolutionary analysis were performed on aligned common codon sequences of *nanA*, *nonA, nanB, nanC, bgaA, or strH* genes. Complete identical sequences were excluded. Whole gene non-synonymous/synonymous (dN/dS) ratio calculations, as well as statistical tests for negative or positive selection for individual codons, were performed using two-rate Fixed Effects Likelihood (FEL) and Fast Unconstrained Bayesian AppRoximation (FUBAR) in the HyPhy software package^44^,^45^. Comparing codon selection between *nanA* and *nonA* genes was performed using LR tests in the HyPhy^44^.

Ancestral states for bacterial sialidases were reconstructed in Mesquite version 3.04^46^ using a parsimony model with characters treated as unordered. The reconstruction was performed on the phylogenetic tree generated by MrBayes. States of active or inactive sialidase were assigned “0” or “1” for each taxon. Unavailable data were coded as missing.

### Bacterial strains and cell lines

Streptococcal strains listed in Supplementary Table 5 were cultured in Todd-Hewitt broth (BD Biosciences) supplemented with or without 0.2% yeast extract (BD Biosciences) (THY or TH medium) at 37 °C. *Streptococcus pseudopneumoniae* ATCC BAA-960 (also called as SK1069 or CCUG 49455)^47^ was kindly provided by Dr. T. Hoshino, Nagasaki University, Japan. The *Escherichia coli* strain TOP10 (Invitrogen) was used as a host for derivatives of plasmids pSET4s, pDCerm, and pDESTerm. All *E. coli* strains were cultured in Luria-Bertani (LB) broth at 37 °C with agitation. For selection and maintenance of mutants, antibiotics were added to the media at the following concentrations: ampicillin (Wako), 100 μg/ml for *E. coli*; kanamycin (Sigma-Aldrich), 50 μg/ml for *E. coli*; chloramphenicol (Sigma-Aldrich), 10 μg/ml for *E. coli*; spectinomycin (Wako), 100 μg/ml for *E. coli* and 150 μg/ml for GBS; and erythromycin (Sigma-Aldrich), 400 μg/ml for *E. coli* and 5 μg/ml for GBS. Human brain endothelial cell line (hBMEC) was maintained in RPMI 1640 medium (Invitrogen) supplemented with 10% FBS, 10% NuSerum (BD), and 1% MEM nonessential amino acids, and were incubated at 37 °C in 5% CO_2_.

### Construction of mutant strain

The construction of in-frame deletion mutants was conducted using a temperature-sensitive shuttle vector, pSET4s, as reported previously^48^,^49^. During the course of construction, a merodiploid strain was created after the first allelic replacement and then resolved to possess either mutant or wild type alleles after the second allelic replacement. To minimize the effect of secondary mutations and epigenetic changes that may have arisen during mutagenesis, a clone possessing the wild type allele was used as a wild-type strain. Both the wild-type and an in-frame deletion mutant strain arose from the same merodiploid ancestor. The correct in-frame deletion of genes was confirmed by site-specific PCR using purified chromosomal DNA. To create NonA and NanA-swapped GBS strain, Δ*nonA*[pNanA], pNanA plasmid was introduced respectively into GBSΔ*nonA* strain by electroporation^14^. pNanA was constructed by ligating *nanA* gene from *S. pneumoniae* strain D39 into pDESTerm plasmid^50^.

### Sialidase activity assay

Sialidase activities of bacterial cells and supernatants were determined by Neuraminidase assay kit (abcam). Streptococcal strains were grown to the mid-log phase (OD_600_ = 0.4–0.5) and centrifuged. To prepare bacterial cell fraction, the bacterial pellet was washed by PBS and resuspended in PBS. The supernatant was used as a supernatant fraction. The samples were incubated for 2 hours at 37 °C and fluorescence intensity was measured at Ex/Em = 350 nm/460 nm.

### Real-time reverse transcription-PCR (RT-PCR) assay

Total RNA of GBS strains grown to the exponential phase (OD_600_ = 0.5) was isolated with RNeasy mini kit and RNase-Free DNase Set (Qiagen). Then, cDNA was synthesized with Transcriptor First Strand cDNA Synthesis Kit (Roche). Real-time RT-PCR analysis was conducted using StepOnePlus Real-Time PCR system (Thermo Fisher Scientific) and KAPA SYBR Fast qPCR Kit (KAPA Biosystems). Data for *gyrA* were used as internal control. Primers are listed in Supplementary Table 6.

### ECA-binding assay

ECA-binding assay was performed as previously described^51^. GBS strains were grown to the mid-log phase and resuspended in PBS to adjust OD_600_ to 0.1. The bacteria were incubated on ice with FITC-conjugated *Erythrina cristagalli* agglutinin (ECA; Vector Laboratories, CA) at 10 μg/mL for 30 min. And then, bacterial cells were washed and resuspended in PBS. The ECA-binding activities on the surface of live bacterial cells were analyzed with a CyFlow SL flow cytometer.

### hBMEC association and invasion assay

The bacterial association to and invasion of hBMEC were quantified with minor modifications as described previously^52^,^53^,^54^. GBS strains were grown to mid-log phase (OD_600_ = 0.5) and resuspended in PBS (OD_600_ = 0.1). hBMECs were seeded at 2 × 10^5^ cells per well in RPMI1640 supplemented with 10% FBS in 24-well plates 1 d prior to bacterial infection. In each well, ~2.0 × 10^6^ CFU of bacteria was added to infect with ~2.0 × 10^5^ hBMECs at an multiplicity of infection (MOI) of 10 in a final volume of 500 μl, and the plate was centrifuged at 1600 rpm for 5 min to initiate their contact. To determine bacterial adhesion, the infected cells were incubated for 1 h, washed three times with PBS, and harvested with a trypsin and 0.025% Triton X-100 solution. The number of bacterial association was quantified by serial dilution plating. To examine bacterial invasion, hBMECs were washed following 1 h-incubation, and 500 μL of media containing 100 μg/mL of gentamicin was added and cells were incubated for an additional 1 h. The cells were washed and lysed, and the number of bacterial invasion was quantified. The bacterial association or invasion rate was calculated by dividing the number of bacterial association/invasion by the number of original inoculums. The invasion rate of bacterial association was also calculated by dividing the number of bacterial invasion by the number of bacterial association.

### Blood bactericidal assay

A blood bactericidal assay was performed as previously described^52^,^55^,^56^. Blood was obtained via venopuncture from healthy donors. It was performed under written informed consent according to a protocol approved by the institutional review boards of Osaka University Graduate School of Dentistry. The GBS cells grown to the mid-log phase were washed and resuspended in PBS, and OD_600_ was adjusted to 0.1. Bacterial cells (10 μl) were combined with fresh human blood (190 μl), and then the mixture was incubated at 37 °C in 5% CO_2_ for 1, 2, and 3 hours. Viable cell counts were determined by plating diluted samples onto THY agar. Growth index was calculated as the number of CFU at the specified time point/number of CFU in the initial inoculum.

### Mice infection assay

All mouse experiments were conducted in accordance with animal protocols approved by the Animal Care and Use Committees at Osaka University Graduate School of Dentistry (24-025-2). CD-1 (ICR: IGS) mice (6 weeks, female; Oriental) were infected with 3.5 × 10^7^ CFU of GBS via the tail vein. After 20 h post-infection, blood aliquots were collected from mice just after general euthanasia. The samples of brain/meninges were collected following perfusion with PBS. Bacterial counts in blood and brain homogenates were determined by plating serial dilutions. Bacterial counts in brain were corrected for differences in each brain weight.

### Statistical analysis

Statistical analysis of *in vitro* and *in vivo* experiments was performed using a nonparametric analysis, Mann-Whitney *U* test. The tests were carried out with Graph Pad prism version 6.0 e (GraphPad Software, Inc.). In evolutionary analysis, *P *< 0.1 was regarded as a significant difference as well as HyPhy default setting.

## Additional Information

**How to cite this article**: Yamaguchi, M. *et al*. Evolutionary inactivation of a sialidase in group B *Streptococcus*. *Sci. Rep.* **6**, 28852; doi: 10.1038/srep28852 (2016).

## Supplementary Material

Supplementary Information

## Figures and Tables

**Table 1:** Evolutionary analyses of *nanA*, *nonA, nanB, nanC, bgaA, and strH* genes.

Gene	Species	Number of Strains	dN/dS	Codons evolving under positive selection	Codons evolving under negative selection
*nanA*	*S. pneumoniae*	16	0.231	0.268% (2/745)	14.362% (107/745)
*nonA*	*S. agalactiae*	16	0.315	0% (0/452)	1.991% (9/452)
*nanB*	*S. pneumoniae*	16	0.309	0% (0/454)	0.881% (4/454)
*nanC*	*S. pneumoniae*	6	0.192	0% (0/740)	2.703% (20/740)
*bgaA*	*S. pneumoniae*	14	0.194	0.313% (7/2233)	5.867% (131/2233)
*strH*	*S. pneumoniae*	17	0.595	0.152% (2/1319)	0.455% (6/1319)

Evolutionary analysis was performed using Baysian inference of aligned *nanA, nonA, nanB, nanC, bgaA, or strH* sequences from complete genomes of *S. pneumoniae* or *S. agalactiae*, with two rate FEL in the HyPhy software package. The dN/dS means ratio of non-synonymous changes to synonymous changes in overall analyzed genes. Individual codons with a statistically significant signature were also calculated and are expressed as a percentage of the total number of codons used in the analysis.

**Table 2:** Comparing codon selection between *nanA* and *nonA* genes.

Tests	LR	DF	*P*-value
The distributions	14.855	10	0.137
Selective regimes (dN/dS and proportions)	9.602	2	0.008
Selection strength (dN/dS)	−0.020	1	1.000
The proportions of codons under selection	8.120	1	0.004

Comparing codon selection was performed using Baysian inference of aligned *nanA* or *nonA* sequences, and distribution comparison tests in the HyPhy software package. LR; Likelihood ratio. DF; degrees of freedom.
