# Supplementary material for: Evolutionary inactivation of a sialidase in group B Streptococcus
Source: Sci Rep. 2016 Jun 29;6:28852. doi: 10.1038/srep28852 (PMC4926279; doi:10.1038/srep28852)
Supplement: Supplementary Information [file srep28852-s1.pdf]

**Supplementary information**

# Evolutionary inactivation of a sialidase in group B *Streptococcus*

Masaya Yamaguchi[a, b, *], Yujiro Hirose[a, c], Masanobu Nakata[a], Satoshi Uchiyama[b], Yuka Yamaguchi[b], Kana Goto[a], Tomoko Sumitomo[a], Amanda L. Lewis[e], Shigetada Kawabata[a], Victor Nizet[b, d, f]

[a]Department of Oral and Molecular Microbiology, Osaka University Graduate School of Dentistry, Suita, Osaka, Japan; [c]Department of Urology, Nagoya University Graduate School of Medicine, Nagoya, Aichi, Japan; [b]Department of Pediatrics, [d]Skaggs School of Pharmacy and Pharmaceutical Sciences, University of California San Diego, La Jolla, CA, USA; [e]Department of Molecular Microbiology and Ob/Gyn, Washington University School of Medicine, St. Louis, Missouri, USA; [f]Rady Children's Hospital, San Diego, CA, USA.

*Address correspondence to Masaya Yamaguchi, yamaguchi@dent.osaka-u.ac.jp

**Supplementary Figure legends**

**Figure S1. Maximum likelihood tree of *nanA* and *nonA* genes.** Bootstrap values are shown near the nodes. Strains with identical DNA sequences are listed on the same branch. The scale bar indicates nucleotide substitutions per site.

**Figure S2. Schematic illustration of domain structures in streptococcal NanA, NanB, and NanC proteins.** Strains with identical sequences are listed on the same branch. *S. iniae* NanA, *S. pneumoniae* NanB and NanC lack an LPXTG motif.

**Figure S3. Sialidase activities of bacterial cells and culture supernatants.** A. The sialidase activities of GBS A909 and streptococcal type strains. B. The sialidase activities of GBS clinical strains and *S. pneumoniae* D39. C. The sialidase activities of clinically isolated *S. iniae* strains. The strains were isolates from brain of a diseased hybrid-stripedbass or tilapia at the Kent SeaTech aquaculture facility in Mecca, CA. After 2 h incubation at 37°C, fluorescence of degraded sialidase substrate was measured with excitation and emission wavelengths of 350 and 460 nm, respectively. Data are presented as the mean of triplicate or sextuplet samples. S.E.

values are represented by vertical lines. The limit of sensitivity of the assay is 0.3 mU/mL.

**Figure S4. Maximum likelihood tree of *nanA, nanB, nanC,* and *nonA* genes.** Bootstrap values are shown near nodes. The scale bar indicates nucleotide substitutions per site.

**Figure S5. Bayesian phylogenetic tree of *nanA* in *S. pneumoniae.*** Percentage of posterior probabilities is shown near the nodes. The scale bar indicates nucleotide substitutions per site. FEL and FUBAR analyses were performed on *nanA* multiple alignment data and this tree.

**Figure S6. Bayesian phylogenetic tree of *nonA* in *S. agalactiae.*** Percentage of posterior probabilities is shown near the nodes. The scale bar indicates nucleotide substitutions per site. FEL and FUBAR analyses were performed on *nonA* multiple alignment data and this tree.

**Figure S7. Bayesian phylogenetic tree of *nanB* in *S. pneumoniae.*** Percentage of posterior probabilities is shown near the nodes. The scale bar indicates nucleotide substitutions per site. FEL and FUBAR analyses were performed on *nanB* multiple alignment data and this tree.

**Figure S8. Bayesian phylogenetic tree of *nanC* in *S. pneumoniae.*** Percentage of posterior probabilities is shown near the nodes. The scale bar indicates nucleotide substitutions per site. FEL and FUBAR analyses were performed on *nanC* multiple alignment data and this tree.

**Figure S9. Bayesian phylogenetic tree of *bgaA* in *S. pneumoniae.*** Percentage of posterior probabilities is shown near the nodes. The scale bar indicates nucleotide substitutions per site. FEL and FUBAR analyses were performed on *bgaA* multiple alignment data and this tree.

**Figure S10. Bayesian phylogenetic tree of *strH* in *S. pneumoniae.*** Percentage of posterior probabilities is shown near the nodes. The scale bar indicates nucleotide substitutions per site. FEL and FUBAR analyses were performed on *strH* multiple alignment data and this tree.

**Figure S11. Expression of *nonA* in GBS A909.** The graph shows fold transcript level of *cylE* and *nonA* in GBS A909 wild type (filled bars) and Δ*nonA* mutant strain (open bars). RNA extractions were performed from mid-log phase cultures. The level of DNA gyrase subunit A gene (*gyrA*) transcription was used as an internal standard. The data represent the mean values

of triplicate samples. S.E. values are represented by vertical lines.

Table S1. Homology between NanA, NanB, and NanC of *S. pneumoniae* D39 and NonA of GBS A909

| | NanA | NonA | NanB | NanC |
|---|---|---|---|---|
| NanA | - | 49/58/75/1 | 63/25/43/17 | 53/27/44/16 |
| NonA | 70/58/75/1 | - | 62/28/45/16 | 60/27/44/15 |
| NanB | 88/25/43/17 | 62/28/45/16 | - | 94/51/69/0 |
| NanC | 70/27/44/16 | 57/27/44/15 | 88/51/69/0 | - |

Cover/Indentities/Positives/Gaps (%)

Table S2. Bacterial strains and the accession number of the genome sequences used for phylogenetic tree

| Bacteria | Strain | Accession number |
|---|---|---|
| *Streptococcus pneumoniae* | D39 | CP000410.1 |
| | JJA | CP000919.1 |
| | CGSP14 | CP001033.1 |
| | Hungary19A-6 | CP000936.1 |
| | ST556 | CP003357.1 |
| | Taiwan19F-14 | CP000921.1 |
| | SPNA45 | CP000936.1 |
| | AP200 | CP002121.1 |
| | G54 | CP001015.1 |
| | gamPNI0373 | CP001845.1 |
| | TCH8431/19A | CP001993.1 |
| | ATCC700669 | FM211187.1 |
| | P1031 | CP000920.1 |
| | 70585 | CP000918.1 |
| | TIGR4 | AE005672.3 |
| | 670-6B | CP002176.1 |
| | NT_110_58 | CP007593.1 |
| *Streptococcus agalactiae* | A909 | CP000114.1 |
| | ILRI112 | HF952106.1 |
| | GBS2-NM | CP007571.1 |
| | GBS6 | CP007572.1 |
| | GBS1-NY | CP007570.1 |
| | SA20-06 | CP003919.1 |
| | 138spar | CP007565.1 |
| | 138P | CP007482.1 |
| | 2-22 | FO393392.1 |
| | GD201008-001 | CP003810.1 |
| | NEM316 | AL766854.1 |
| | NGBS572 | CP007632.1 |
| | 2603V/R | NC_004116.1 |
| | ILRI005 | HF952105.1 |
| | CNCTC 10/84 | CP006910.1 |
| | 09mas018883 | HF952104.1 |
| | NGBS061 | CP007631.2 |
| | COH1 | HG939456.1 |
| *Streptococcus iniae* | ISNO | CP007587.1 |
| | ISET0901 | CP007586.1 |
| | YSFST01-82 | CP010783.1 |
| | SF1 | CP005941.1 |
| *Streptococcus intermedius* | B196 | CP003857.1 |
| | C270 | CP003858.1 |
| | JTH08 | AP010969.1 |
| *Streptococcus mitis* | B6 | FN568063.1 |
| | KCOM 1350 | CP012646.1 |
| *Streptococcus oralis* | Uo5 | FR720602.1 |
| *Streptococcus pseudopneumoniae* | IS7493 | CP002925.1 |
| *Streptococcus sp.* | VT 162 | CP007628.2 |
| *Erysipelothrix rhusiopathiae* | SY1027 | AP012027.1 |
| | Fujisawa | CP005079.1 |

Table S3. The frameshift mutation in GBS *nonA* genes.

| GBS strain | DNA sequence (5’–3’) |
|---|---|
| A909 | T GAT TGG GGA AAC ATA GGA ATG GTT ATT CGC CGT AGT GA |
| SA20-06 | T GAT TGG GG<u>**G**</u> AAA CAT AGG AAT GGT TAT TCG CCG TAG TGA |
| GX026 | T GAT TGG GG<u>**G**</u> AAA CAT AGG AAT GGT TAT TCG CCG TAG TGA |
| 2-22 | T GAT TGG GG<u>**G**</u> AAA CAT AGG AAT GGT TAT TCG CCG TAG TGA |
| 138spar | T GAT TGG GG<u>**G**</u> AAA CAT AGG AAT GGT TAT TCG CCG TAG TGA |
| 138P | T GAT TGG GG<u>**G**</u> AAA CAT AGG AAT GGT TAT TCG CCG TAG TGA |

Bold and underlined character is the inserted nucleotide. Box shows stop codons generated by the framshift.

Table S4. Evolutionary analyses of *nanA*, *nonA, nanB, nanC, bgaA,* and *strH* genes

| Gene | Species | Number of Strains | dN/dS | Codons evolving under positive selection | Codons evolving under negative selection |
|---|---|---|---|---|---|
| *nanA* | *S. pneumoniae* | 16 | 0.29 | 0.940% (7/745) | 15.302% (114/745) |
| *nonA* | *S. agalactiae* | 16 | 0.35 | 0.885% (4/452) | 1.770% (8/452) |
| *nanB* | *S. pneumoniae* | 16 | 0.35 | 0.441% (2/454) | 0.661% (3/454) |
| *nanC* | *S. pneumoniae* | 6 | 0.29 | 0% (0/740) | 2.703% (20/740) |
| *bgaA* | *S. pneumoniae* | 14 | 0.29 | 0.806% (18/2233) | 4.881% (109/2233) |
| *strH* | *S. pneumoniae* | 17 | 0.50 | 0.758% (10/1319) | 0.607% (8/1319) |

Evolutionary analysis was performed using Baysian inference of aligned *nanA, nonA, nanB, nanC, bgaA,* and *strH* sequences from *S. pneumoniae* or *S. agalactiae*, with FUBAR in the HyPhy software package. The dN/dS means ratio of non-synonymous changes to synonymous changes in overall analyzed genes. Individual codons with a statistically significant signature were also calculated and are expressed as a percentage of the total number of codons used in the analysis.

Table S5. Bacterial strains used in sialidase activity assay

| Bacteria | Strain | Remarks |
|---|---|---|
| *Streptococcus pneumoniae* | D39 | Serotype 2 |
| *Streptococcus agalactiae* | A909 | Serotype Ia |
| | M709 | Serotype Ib |
| | GBS2-NM | Serotype II |
| | GBS6 | Serotype III |
| | GBS1-NY | Serotype V |
| | SA20-06 | Serotype VI |
| *Streptococcus mitis* | ATCC 49456/NCTC 12261/SK142 | Type strain |
| *Streptococcus oralis* | ATCC 35037/NCTC 11427 | Type strain |
| *Streptococcus pseudopneumoniae* | ATCC BAA-960/CCUG 49455/SK1069 | Type strain |
| *Streptococcus intermedius* | ATCC 27335/SK54 | Type strain |
| *Streptococcus iniae* | K122 | Clinical strains |
| | K139 | Clinical strains |
| | K277 | Clinical strains |
| | K288 | Clinical strains |
| | K375 | Clinical strains |
| | K436 | Clinical strains |
| | K540 | Clinical strains |
| | 94-426 | Clinical strains |

Table S6. Real-time RT-PCR Primers used in this study

| Primers | Sequence (5’ to 3’) |
|---|---|
| A909nonAF | GTGATTGGGGAAACATAGGAATGG |
| A909nonAR | CGATTTGGGTATATTCTTTTTCAG |
| A909cylEF | ATTGAAAATCCCTTGGAAGAGGAGACAG |
| A909cylER | GTTAGGCTAGGGTGAGCCCTCG |
| A909gyrAF | CCACATGGTGATTCATCTATTTAC |
| A909gyrAR | CGCTGGTAAAACAAGAGGTTCACG |

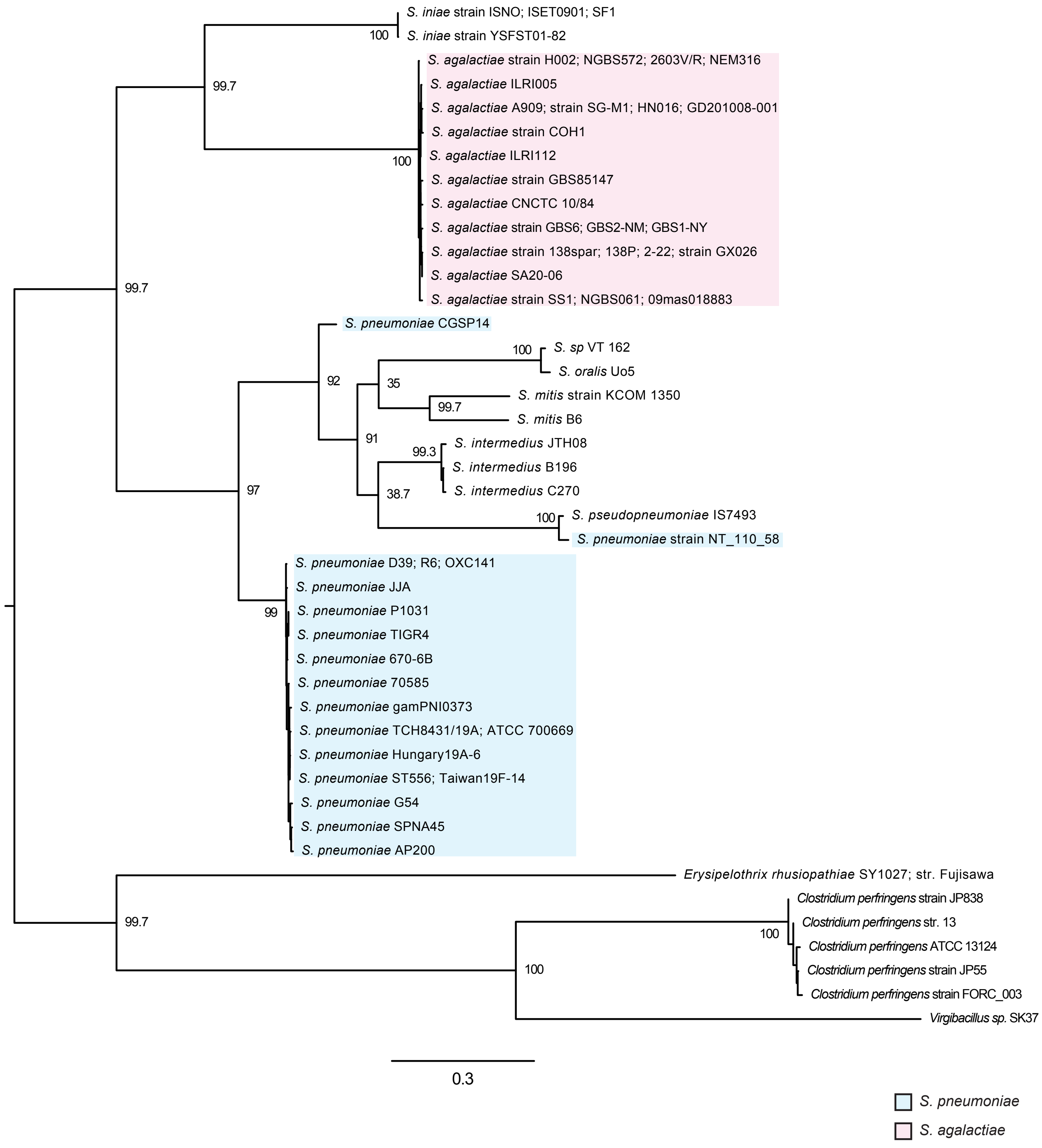


Supplementary Figure 1. Yamaguchi *et al*.

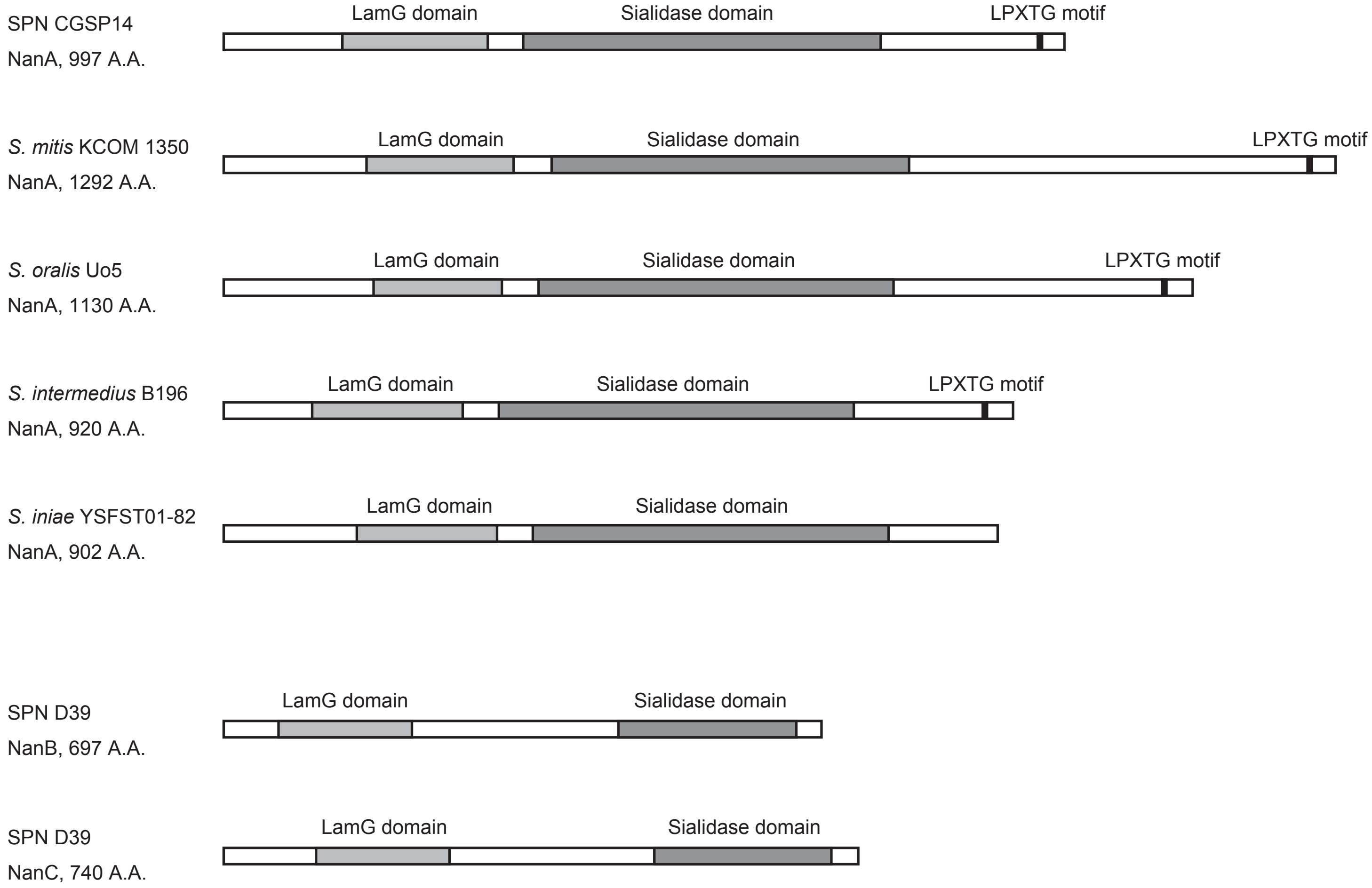


Supplementary Figure 2. Yamaguchi *et al*.

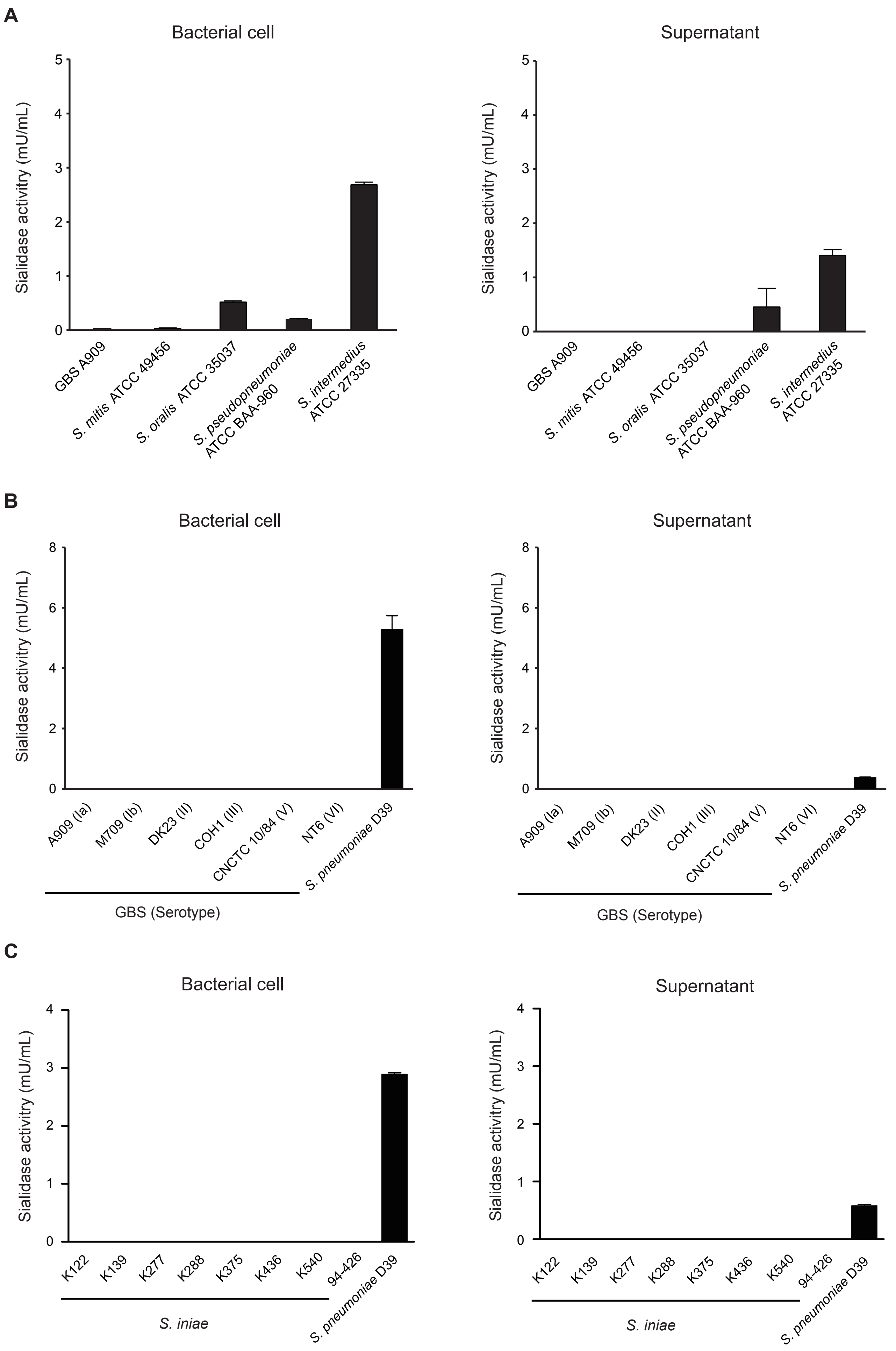


Supplementary Figure 3. Yamaguchi *et al*.

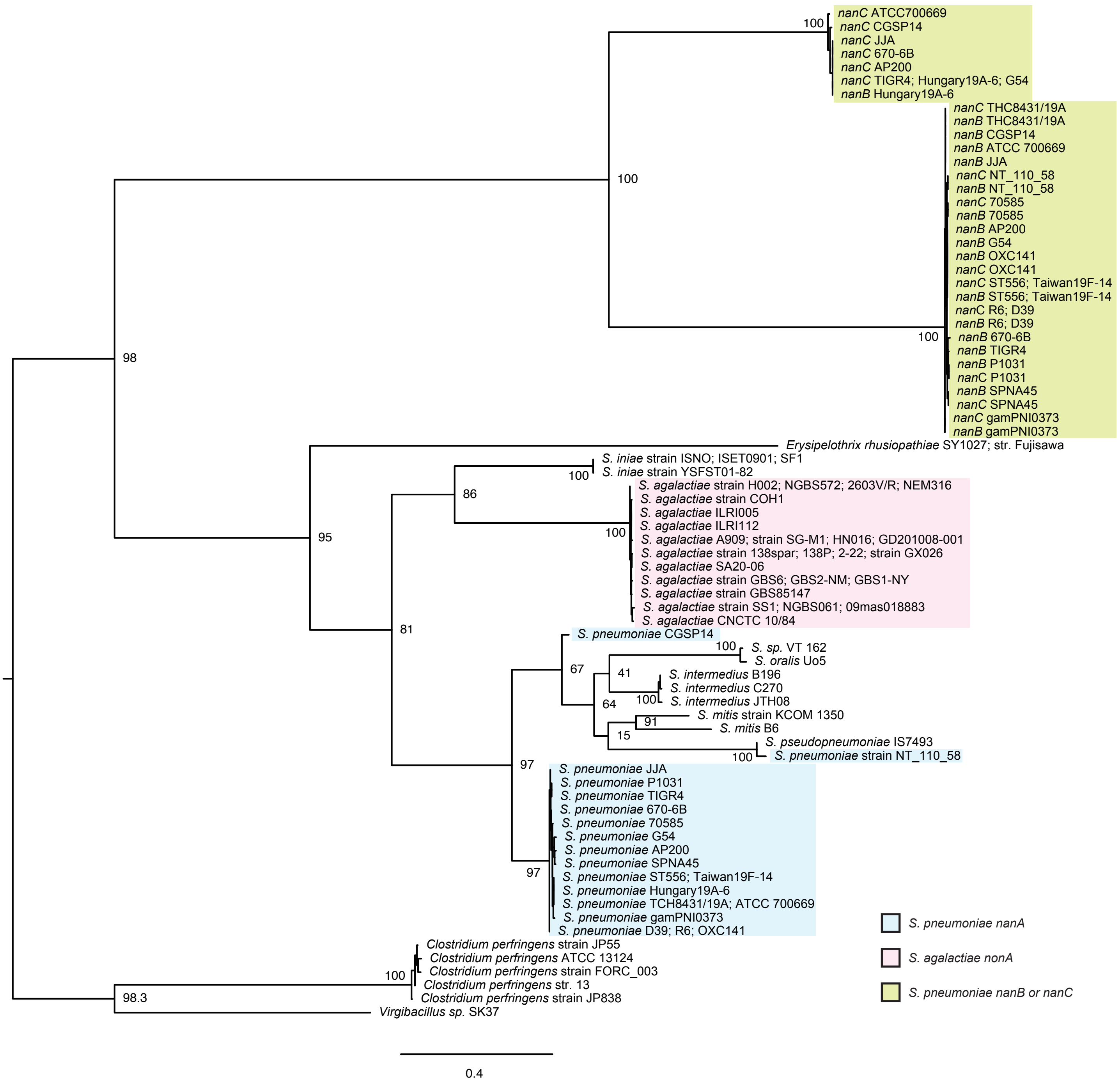


Supplementary Figure 4. Yamaguchi *et al*.

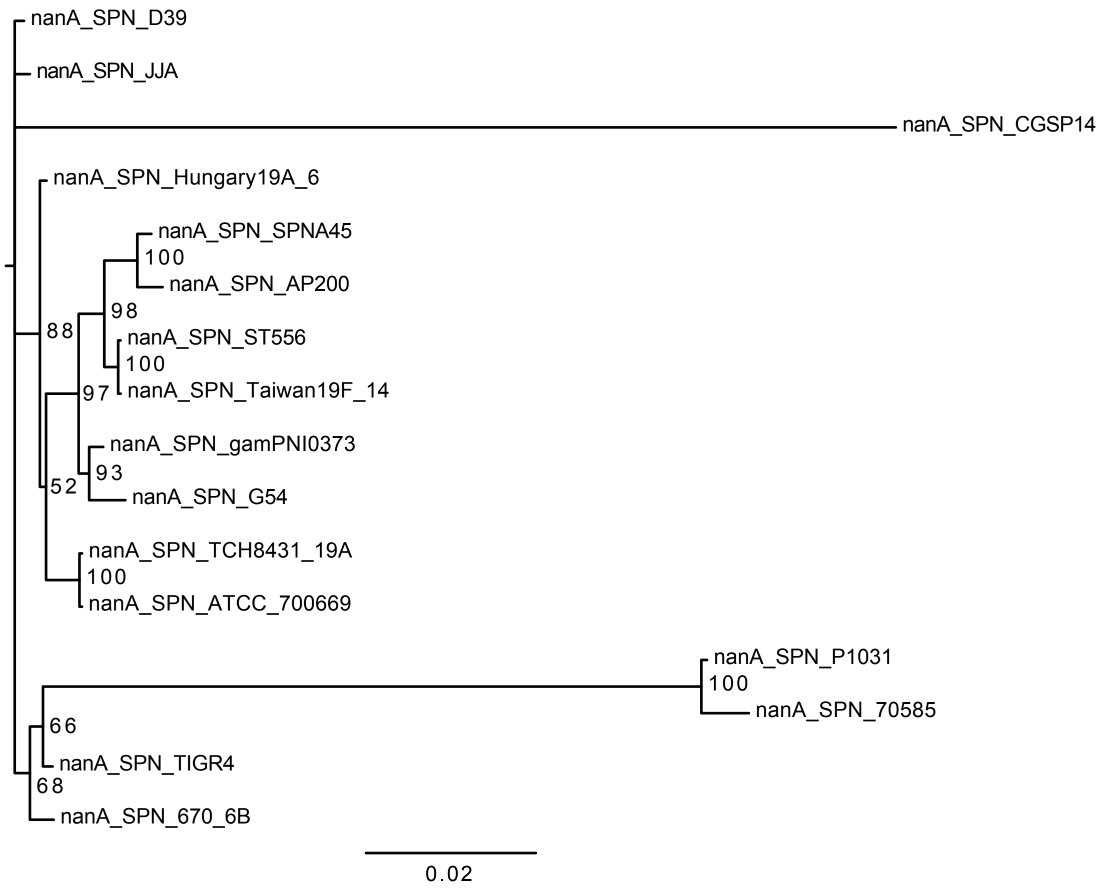


Supplementary Figure 5. Yamaguchi *et al*.

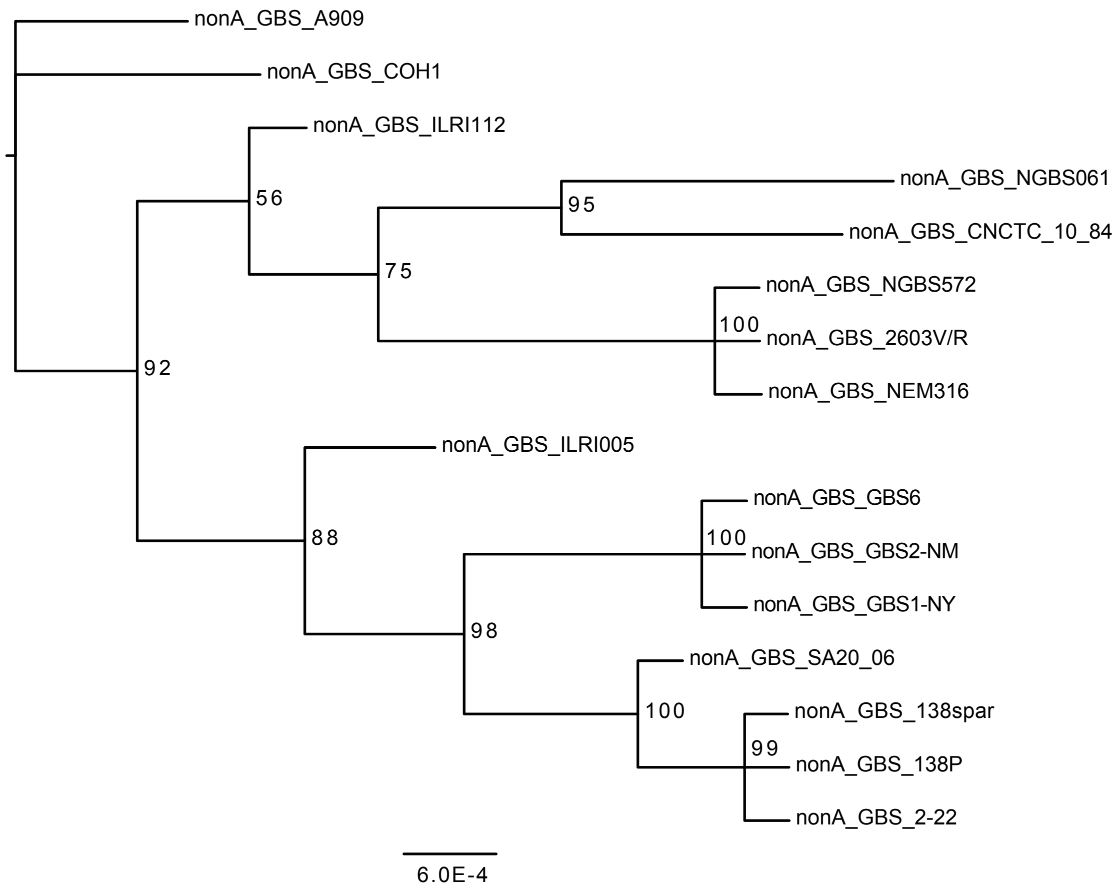


Supplementary Figure 6. Yamaguchi *et al*.

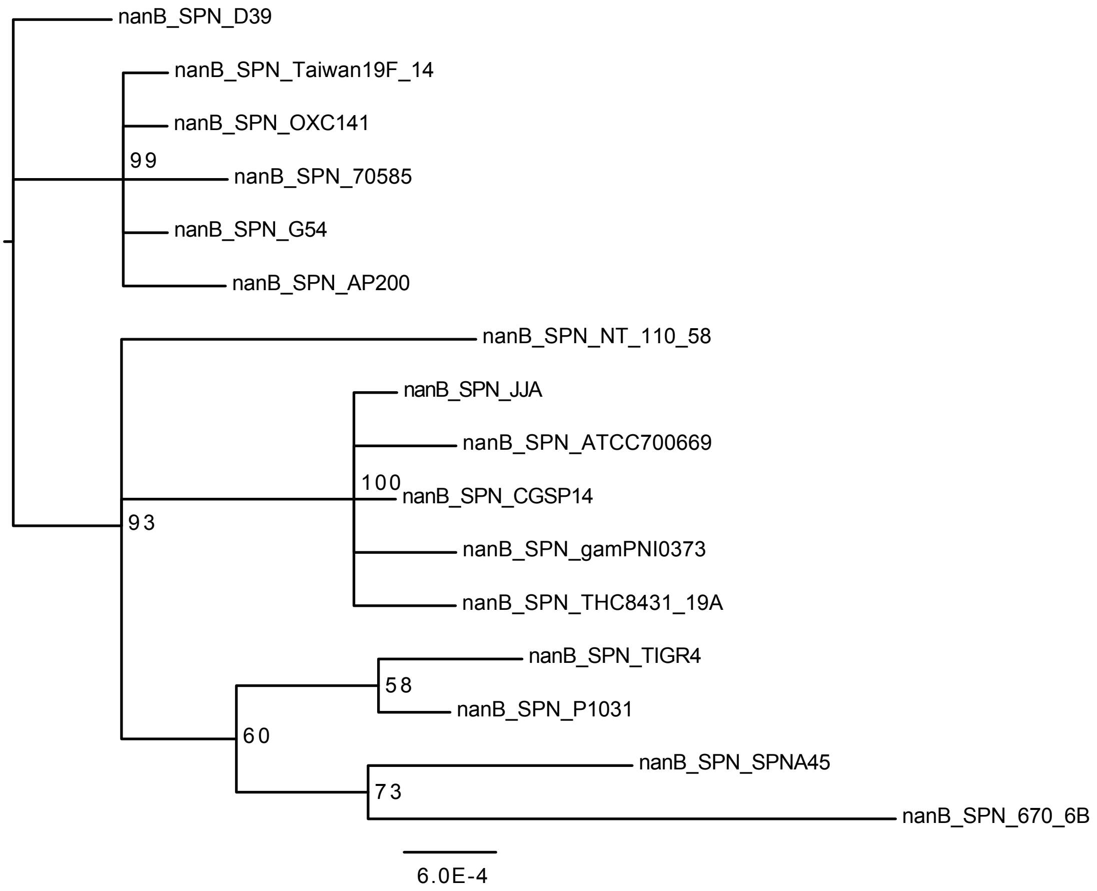


Supplementary Figure 7. Yamaguchi *et al*.

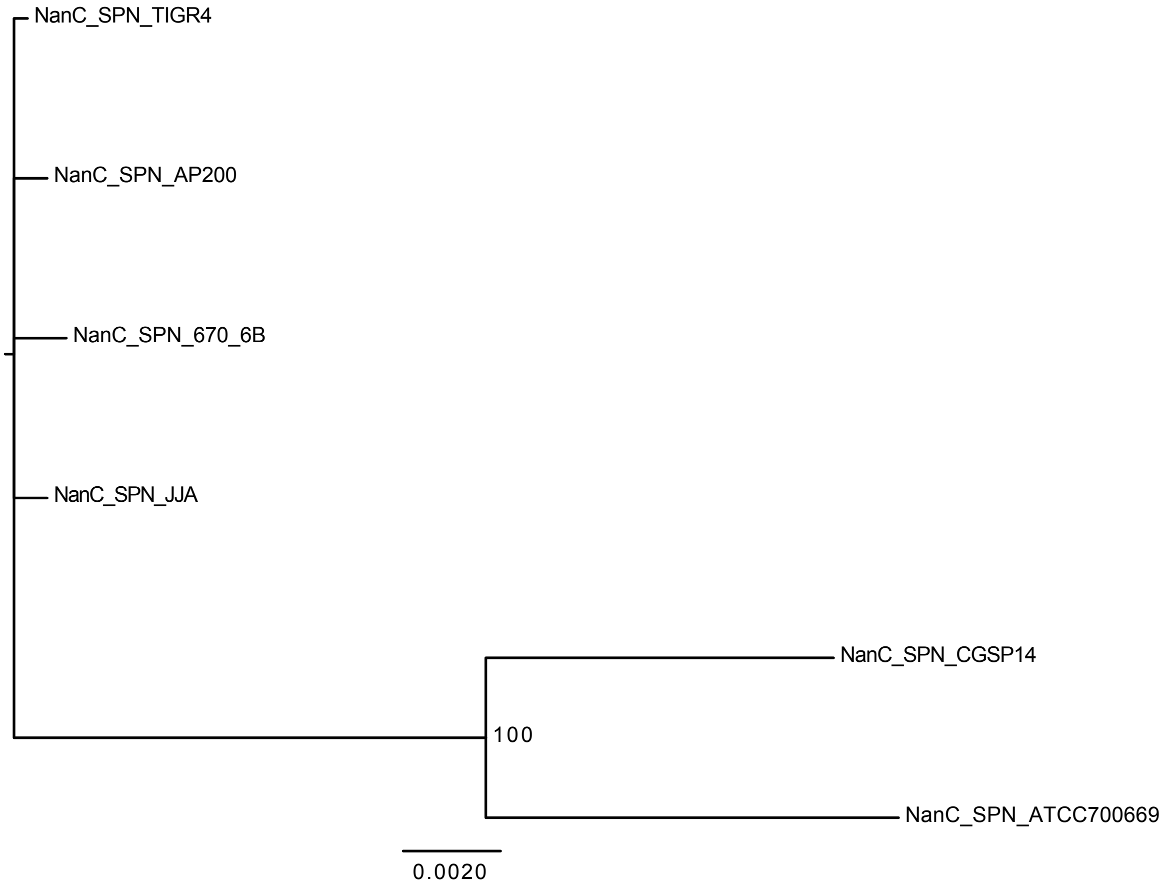


Supplementary Figure 8. Yamaguchi *et al*.

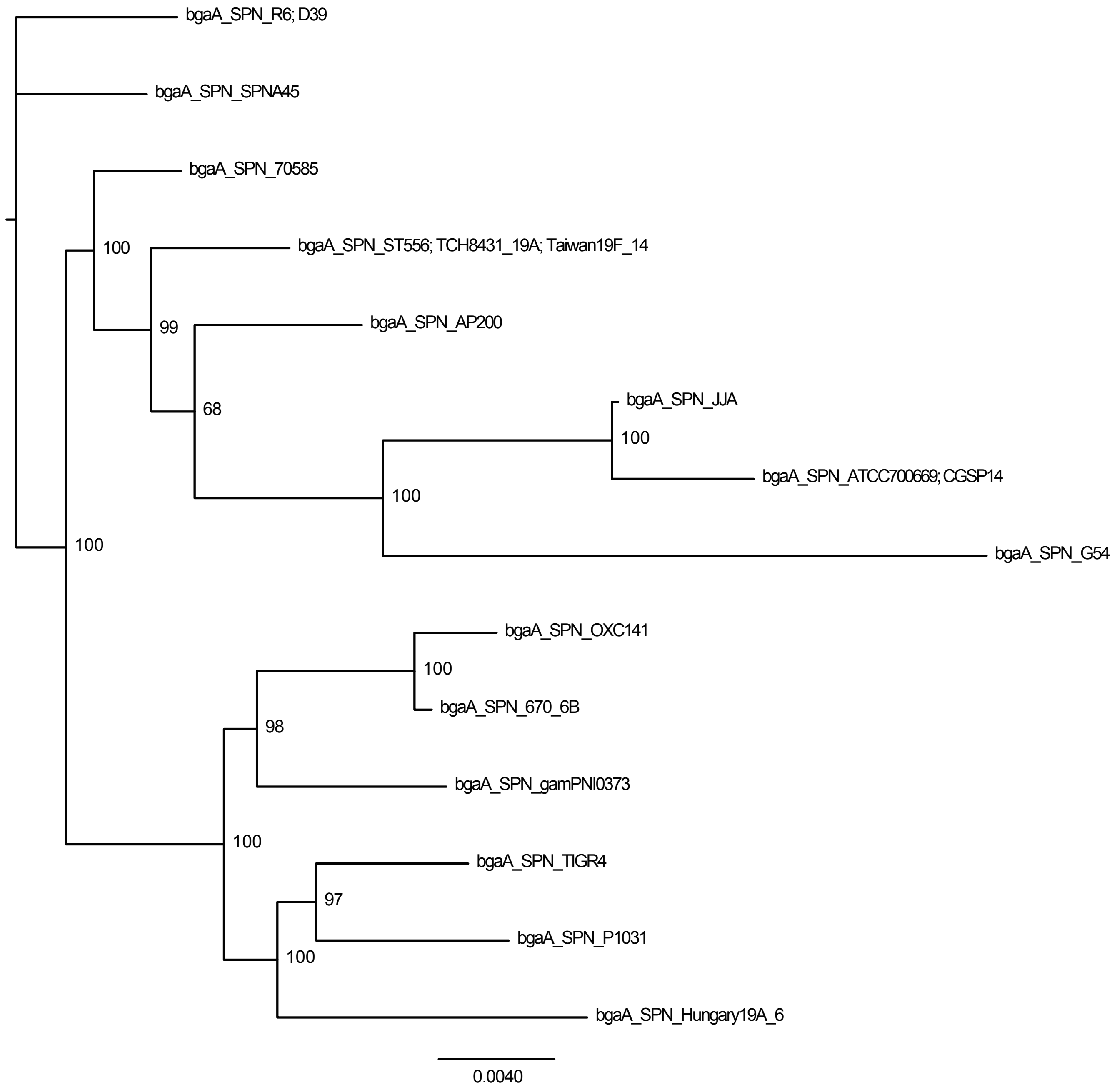


Supplementary Figure 9. Yamaguchi *et al*.

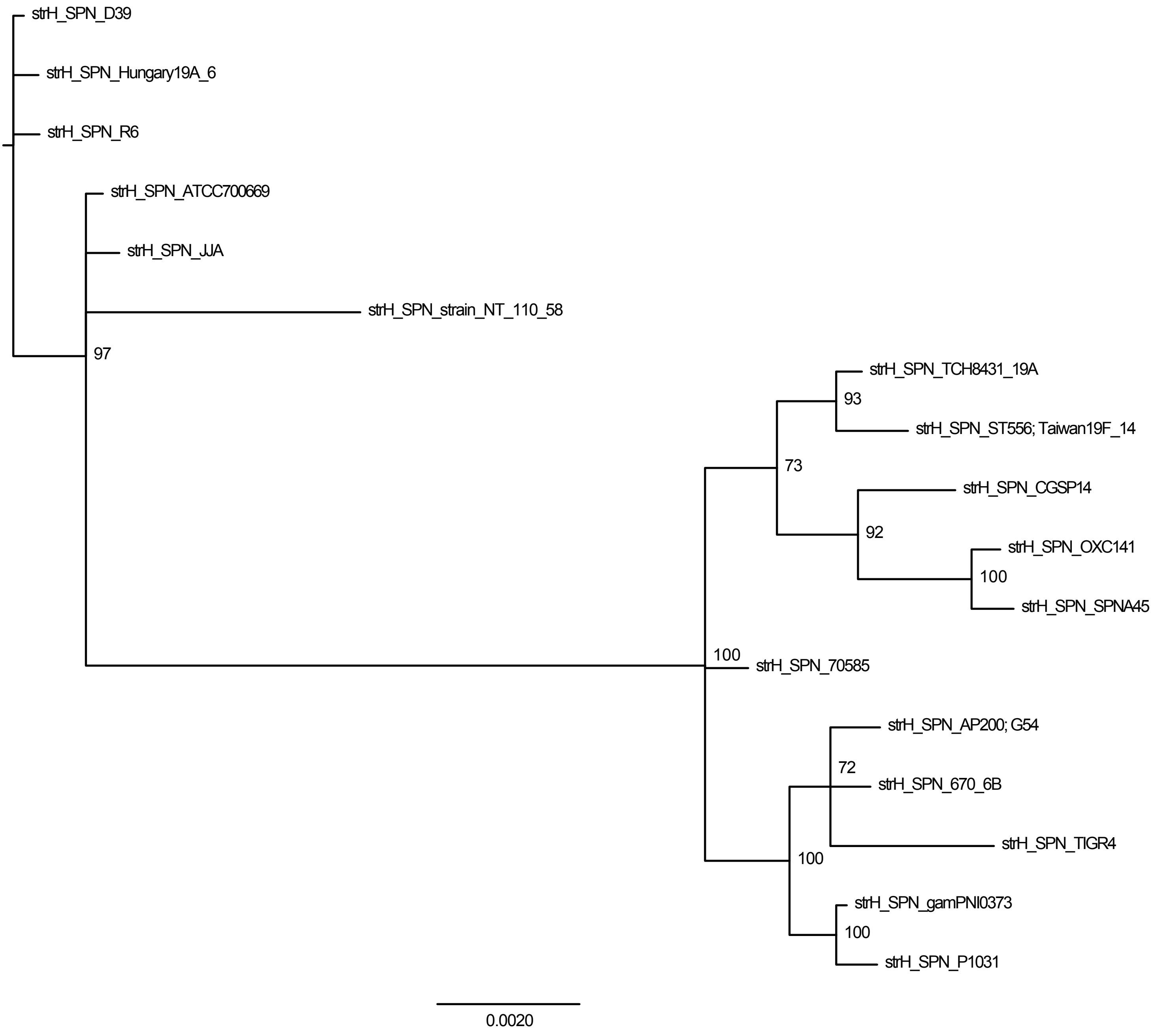


Supplementary Figure 10. Yamaguchi *et al*.

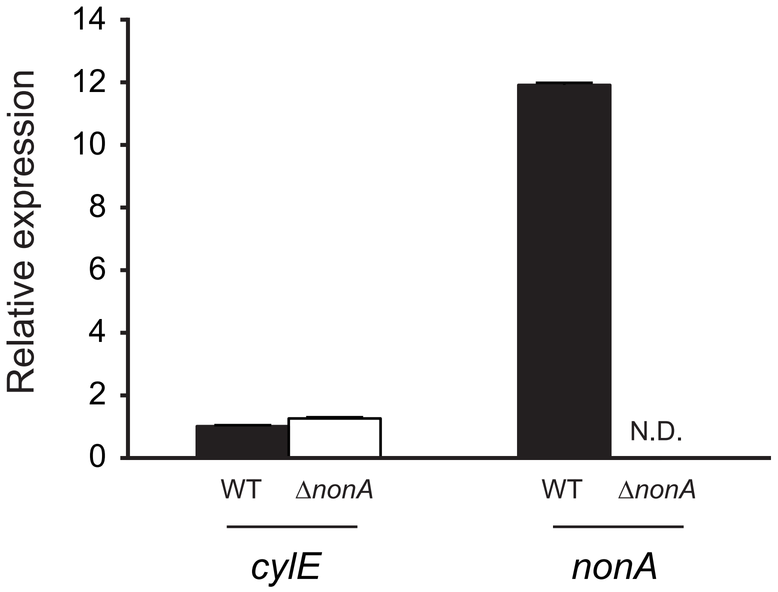


Supplementary Figure 11. Yamaguchi *et al*.
